# Intestinal Cell Kinase Is a Novel Participant in Intestinal Cell Signaling Responses to Protein Malnutrition

**DOI:** 10.1371/journal.pone.0106902

**Published:** 2014-09-03

**Authors:** David T. Bolick, Tufeng Chen, Luís Antonio O. Alves, Yixin Tong, Di Wu, Linwood T. Joyner, Reinaldo B. Oriá, Richard L. Guerrant, Zheng Fu

**Affiliations:** 1 Department of Medicine, Center for Global Health, Digestive Research Center of Excellence, University of Virginia, Charlottesville, Virginia, United States of America; 2 Department of Gastrointestinal Surgery, The Third Affiliated Hospital of Sun Yat-sen University, Guangdong, China; 3 Gastrointestinal Surgery Center, Tongji Hospital, Huazhong University of Science & Technology, Hubei, China; University Claude Bernard Lyon 1, France

## Abstract

Nutritional deficiency and stress can severely impair intestinal architecture, integrity and host immune defense, leading to increased susceptibility to infection and cancer. Although the intestine has an inherent capability to adapt to environmental stress, the molecular mechanisms by which the intestine senses and responds to malnutrition are not completely understood. We hereby report that intestinal cell kinase (ICK), a highly conserved serine/threonine protein kinase, is a novel component of the adaptive cell signaling responses to protein malnutrition in murine small intestine. Using an experimental mouse model, we demonstrated that intestinal ICK protein level was markedly and transiently elevated upon protein deprivation, concomitant with activation of prominent pro-proliferation and pro-survival pathways of Wnt/β-catenin, mammalian target of rapamycin (mTOR), mitogen-activated protein kinase (MAPK), and protein kinase B (PKB/Akt) as well as increased expression of intestinal stem cell markers. Using the human ileocecal epithelial cell line HCT-8 as an *in* *vitro* model, we further demonstrated that serum starvation was able to induce up-regulation of ICK protein in intestinal epithelial cells in a reversible manner, and that serum albumin partially contributed to this effect. Knockdown of ICK expression in HCT-8 cells significantly impaired cell proliferation and down-regulated active β-catenin signal. Furthermore, reduced ICK expression in HCT-8 cells induced apoptosis through a caspase-dependent mechanism. Taken together, our findings suggest that increased ICK expression/activity in response to protein deprivation likely provides a novel protective mechanism to limit apoptosis and support compensatory mucosal growth under nutritional stress.

## Introduction

Intestinal luminal nutrients constitute the primary stimulus for intestinal growth. Intra-lumen food is capable of stimulating gut mucosal growth either directly through local effect at the site of absorption or indirectly by regulating the release of gut hormones that are important for mucosal growth and repair [1], [2]. Starvation is able to cause mucosal atrophy in the small intestine, characterized by diminished intestinal functions as well as altered morphological structures including decreased villous height, crypt depth, surface area, and epithelial cell numbers [3], [4]. In response to a nutrient challenge, the small intestine exhibits a remarkable capacity of mucosal adaptation to prevent atrophy and maintain normal mucosal architecture and functions. However, very little is known about the molecular basis underlying the intestinal cellular responses to nutritional stress. Major signaling pathways such as Wnt/β-catenin [5], PI3K/Akt [6], mTOR/S6K1 [7], and MAPKs [8] govern intestinal cell growth, differentiation, migration and survival in the intestinal mucosa. An intriguing question that has not been fully addressed is whether and how these crucial signaling cascades respond to nutritional deficiency.

Intestinal cell kinase (ICK) is a newly emerged key component in the intestinal cell signaling network [9], [10]. ICK, named after its cloning origin the intestine, is an evolutionarily conserved serine/threonine protein kinase in the protein kinome that is closely related to mitogen-activated protein kinases (MAPKs). In the small intestine, ICK mRNA specifically localizes to the crypt region where intestinal stem/progenitor cells and the rapidly replicating transit-amplifying cells reside, implicating an important role for ICK in the regulation of epithelial cell replication and stem cell activities [9]. To support this hypothesis, we have shown that knockdown of ICK expression *in* *vitro* is able to significantly impair intestinal epithelial cell proliferation [10]. Murine ICK gene encodes a protein of 629 amino acid residues, comprised of a highly conserved N-terminal catalytic domain and a unique long C-terminal domain [11], [12]. ICK can be activated by an upstream kinase CCRK (cell cycle-related kinase) through phosphorylation of the essential Thr-157 residue in its MAPK-like TDY motif [11]. The signaling axis of CCRK-ICK plays an important role in the regulation of cell cycle progression at G1 [10], [13], [14]. However, unlike MAPKs, ICK activity was not acutely stimulated by serum or growth factors [9]. It remains a major question as to what upstream stimuli or environmental cues that may regulate ICK expression and/or activity. The physiologic functions and substrates of ICK in the intestine are still elusive.

We hereby report that nutritional stress as an environmental cue is capable of acutely and transiently regulating ICK protein/activity level. By knocking down ICK expression *in* *vitro* using lentiviral short-hairpin RNA interference (shRNA), we demonstrated that ICK signaling is important for intestinal cell proliferation and survival through β-catenin-mediated and caspase-dependent pathways, respectively. These results suggest that intestinal epithelial cells may up-regulate ICK signaling pathway as a protective mechanism to limit apoptosis and promote compensatory growth during intestinal adaptation to protein malnutrition.

## Materials and Methods

### Animals, Human Cell Lines and Ethics Statement

Animal experiments were carried out according to NIH Animal Welfare Guidelines after approval by the University of Virginia Institutional Animal Care and Use Committee. C57BL/6 mice were purchased from Charles River Laboratories, Inc. Mice were euthanized in a CO_2_ chamber. Human cell lines were purchased from American Culture Type Collection (ATCC) and used in our study after approval by the University of Virginia Institutional Bio-safety Committee.

### Protein Malnutrition Animal Model

Mice were acclimated, fed a regular diet for 7 days, and then assigned to experimental groups matched for body weight. At postnatal day 28, mice assigned to the nourished groups received regular chow containing 20% protein whereas mice assigned to the malnourished groups received isocaloric chow containing 2% protein (Harland Labs).

### *In Vitro* Malnutrition Model

The human ileocecal adenocarcinoma HCT-8 cells [15] obtained from ATCC were cultured in Dulbecco’s modified Eagle’s medium (DMEM) containing 2% glutamine and 10% fetal bovine serum (FBS). For starvation, HCT-8 cells were cultured in DMEM containing 2% glutamine and 0.2%–1% FBS.

### Tissue and Cell Extracts Preparation

After rapid dissection of the mouse intestinal tube, intestinal segments were snap-frozen in liquid nitrogen and stored at –80°C, as previously described [16]. Frozen tissues were grinded into fine powders on dry-ice and lysed in RIPA buffer (20 mM Tris, pH 7.5, 150 mM NaCl, 1% Nonidet P-40, 0.5% sodium deoxycholate, 1 mM EDTA, 0.1% SDS) containing protease inhibitors cocktail (Roche) and phosphatase inhibitors (1 mM sodium orthovanadate, 5 mM sodium fluoride, 1 µM microcystin LR, and 5 mM β-glycerophosphate). After serum starvation, HCT-8 cells were lysed directly in RIPA buffer containing protease and phosphatase inhibitors. Tissue and cell lysates were cleared by centrifugation, and the supernatant was saved frozen until use for Western blot analysis.

### Antibodies

A rabbit polyclonal antibody was raised against the C-terminal peptide (residues 388–400) of mouse ICK, as described in [10]. A rabbit polyclonal antibody was raised against the C-terminal peptide (residues 319–332) of mouse CCRK and used in this study (Genscript). Active β-catenin antibody (clone 8E7) was from Millipore. Anti-GPCR GPR49 (Lgr5) antibody (clone EPR3065Y) was from Abcam. All other antibodies were obtained from Cell Signaling Technology.

### Western Blot

Protein extracts were mixed with an equal volume of 2X Laemmli sample buffer, boiled for 5 min, loaded on a SDS-gel, and then transferred to a PVDF membrane for Western blot, as detailed previously in [16]. After blocking for one hour in 5% dry milk, the membrane was incubated with primary antibody (1–2 µg) in TBS containing 0.1% Tween-20 and 5% bovine serum albumin for 90 min at room temperature followed by extensive rinses and one-hour incubation with horseradish peroxidase (HRP)-conjugated secondary antibody (1∶10,000). Chemiluminescence signals were developed using Millipore Immobilon ECL reagents.

### Lentiviral Short-hairpin RNA Interference

The MISSION TRC ICK shRNA Target Set and the control non-targeting shRNA vector were obtained from Sigma, as described in [10]. Lentivector particles were generated in HEK293T cells as described in [10], [11]. Exponentially growing HCT-8 cells at about 50–60% confluence were infected with lentiviruses expressing either the ICK shRNA or the control shRNA for overnight (12–14 hr) before change of medium. Twenty four hours after infection, cells were plated at about 6–8×10^5^ cells/ml in 10 cm dishes and grown for three to four days in the presence of 5 µg/ml puromycin. Infected cells were harvested for either determination of viable cell number and apoptosis or for protein extraction.

### Determination of Viable Cell Number

Three to four days after infection, unattached cells floating in culture medium and attached cells removed from tissue culture plates by Trypsin-EDTA were pooled and counted using a hemocytometer based on Trypan blue exclusion. The mean cell count of ICK shRNA treated cell culture was plotted as the percentage of the mean cell count of the control shRNA treated cell culture.

### Apoptosis Assay by Annexin V Flow Cytometry

Infected HCT-8 cells were pelleted by centrifugation at 300×g for 5 minutes, washed once with cold PBS, and resuspended in binding buffer. Cells were stained with Annexin V antibody and propidium iodide for 20 minutes according to manufacturer’s instructions (BD Biosciences). After gating on the single cell population, 150,000 cells per sample were analyzed. Samples were analyzed at the University of Virginia Flow Cytometry Core using a Becton Dickinson FACSCalibur instrument.

### Statistical Analysis

Statistical significance of experimental results was analyzed by Student’s *t*-test. Data were presented as means ± standard error. P-values less than 0.05 were considered as significant.

## Results

### Protein malnutrition induced robust and transient up-regulation of key signaling pathways related to intestinal cell proliferation, growth, and survival

In order to gain mechanistic insights into intestinal cell signaling responses and adaptation to nutritional stress, we used a previously established *in* *vivo* animal model to assess cell signaling responses to protein malnutrition [17]. Weaned C57BL6 mice were fed with either a regular diet (20% protein) or an isocaloric low-protein diet (2% protein) for a period of 5 days. Intestinal tissues were harvested at various time points during protein starvation and assessed for the status of major cell signaling pathways (Wnt/β-catenin, mTOR/S6K1, MAPK, and PI3K/Akt) that are important for cell proliferation, growth, and survival in the intestinal mucosa.

**Figure 1:**
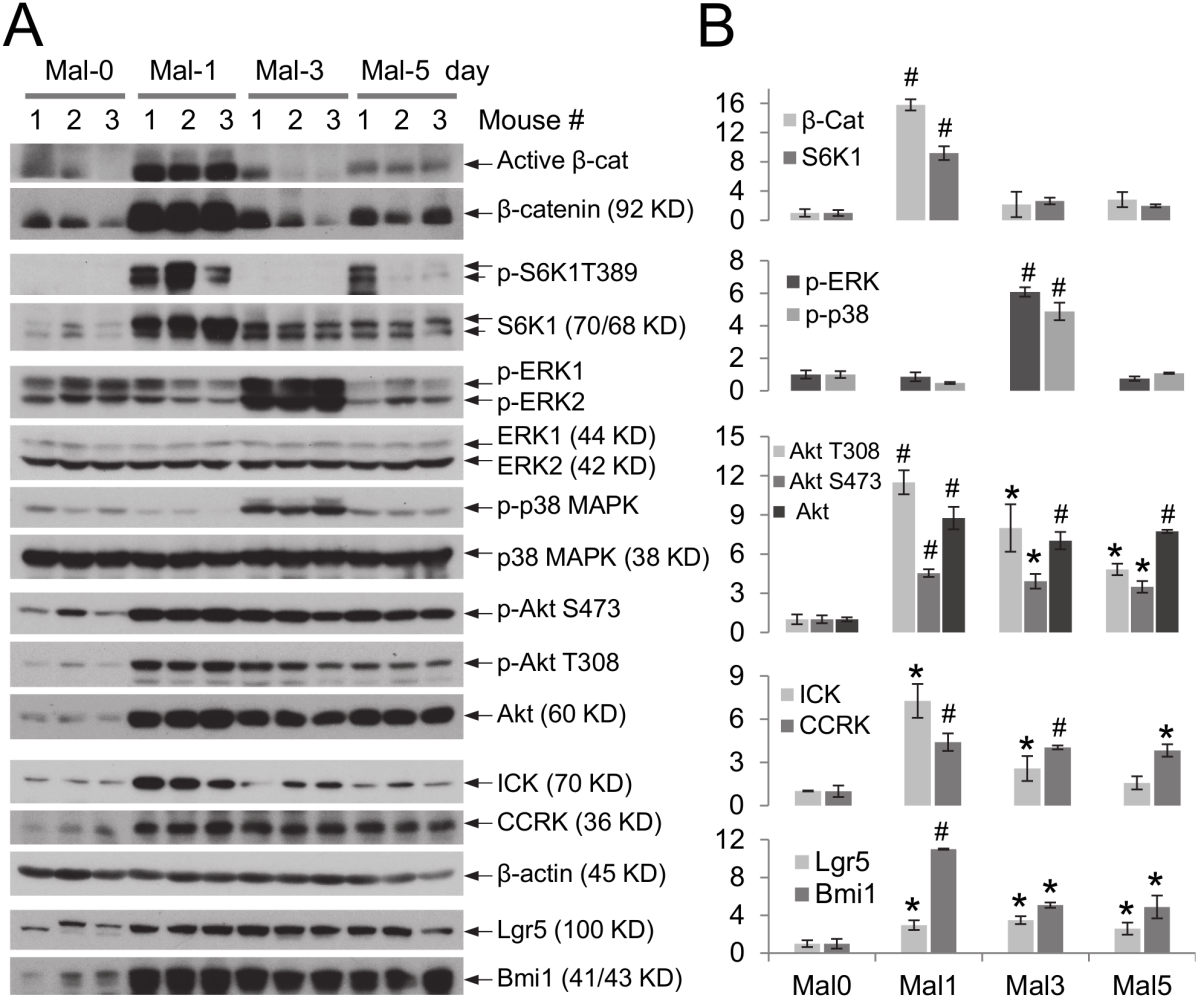
Protein malnutrition induces up-regulation of key signaling pathways that are related to intestinal cell growth and survival. C57BL/6 mice at postnatal day 28 were fed with an isocaloric low-protein (2% protein) diet as compared with the regular diet containing 20% protein for a period of 5 days. (A) Equal amount of total proteins from ileum were Western blotted against antibodies recognizing key components in various signaling pathways as indicated as well as intestinal stem cell markers Lgr5 and Bmi1. The β-actin signal indicates equal loading of total proteins from intestinal tissue extracts. The doublet bands recognized by the S6K1 antibody may represent two alternatively spliced isoforms. (B) After densitometry quantification and normalization against β-actin, the fold change of the protein level relative to the control day zero was shown as mean ± SE, n = 3, *P<0.05, ^#^P<0.01. Similar results were obtained from three independent experiments.

Cytoplasmic β-catenin lacking phosphorylation at Ser33/37/Thr41 (active β-catenin) is stable and serves as the key downstream effector of the Wnt signal by activating transcription after its nuclear translocation. S6K1 is a key downstream target of mTOR that regulates cell growth and survival. At 24 hours of protein malnutrition, both β-catenin and mTOR pathways in the small intestine were markedly up-regulated, as determined by elevated levels of active β-catenin protein and phospho-S6K1 Thr-389 (mTOR target site) signals (Fig. 1). By day 3 of malnutrition, the signaling activities of β-catenin and mTOR pathways were essentially reduced to the basal level. In comparison, activation of MAPK pathways, indicated by augmented phosphorylation of ERK and p38 MAPK in their activation loops, were not evident until day 3 post protein malnutrition. By day 5, these phospho-MAPK signals were decreased to the basal level. For the Wnt/β-catenin and mTOR/S6K1 pathways, changes induced by the low-protein diet occurred primarily at the protein level. In comparison, the alterations in the MAPK pathway in response to protein malnutrition occurred mainly at the phosphorylation-dependent activity level.

The status of the major cell survival pathway PI3K/Akt was also assessed during protein malnutrition (Fig. 1). A significant increase in the total Akt protein level was induced after 24 hours and persisted until day 5 of malnutrition. Activation of Akt/PKB can be regulated through Thr-308 phosphorylation by PDK1 and Ser-473 phosphorylation by mTORC2 [18]. In response to protein starvation, elevated phospho-Akt S473 signal was evident at day 1 and persisted until day 5 of malnutrition, whereas elevated phospho-Akt S308 signal gradually attenuated toward the baseline from day 1 to day 5 of malnutrition. Together, these data suggest that small intestine responded to protein malnutrition by activating major cell proliferation and survival pathways through up-regulation of key signaling components at the protein abundance and/or activity level.

### Protein malnutrition triggered a marked, albeit transient, increase in ICK expression and activity, concurrent with elevated expression of intestinal stem cell markers

ICK is a novel component of the intestinal cell signaling network [9]. *In vitro* evidence implicated a supporting role for ICK in intestinal epithelial cell proliferation [10]. The upstream stimulus for ICK expression or activity is completely unknown. Intriguingly, ICK protein level was significantly increased at 24 hours after protein malnutrition, concomitant with elevated expression of intestinal stem cell markers (Lgr5 [19] and Bmi1 [20]) (Fig. 1). However, this increase was greatly attenuated by day 3 and abolished by day 5 of malnutrition. Similar results were shown for CCRK, the upstream activating kinase for ICK, suggesting that both ICK expression and activity can be regulated by protein nutrient status in the small intestine.

### A significant increase in the ICK protein level in HCT-8 cells can be acutely induced by serum starvation in a reversible manner

**Figure 2:**
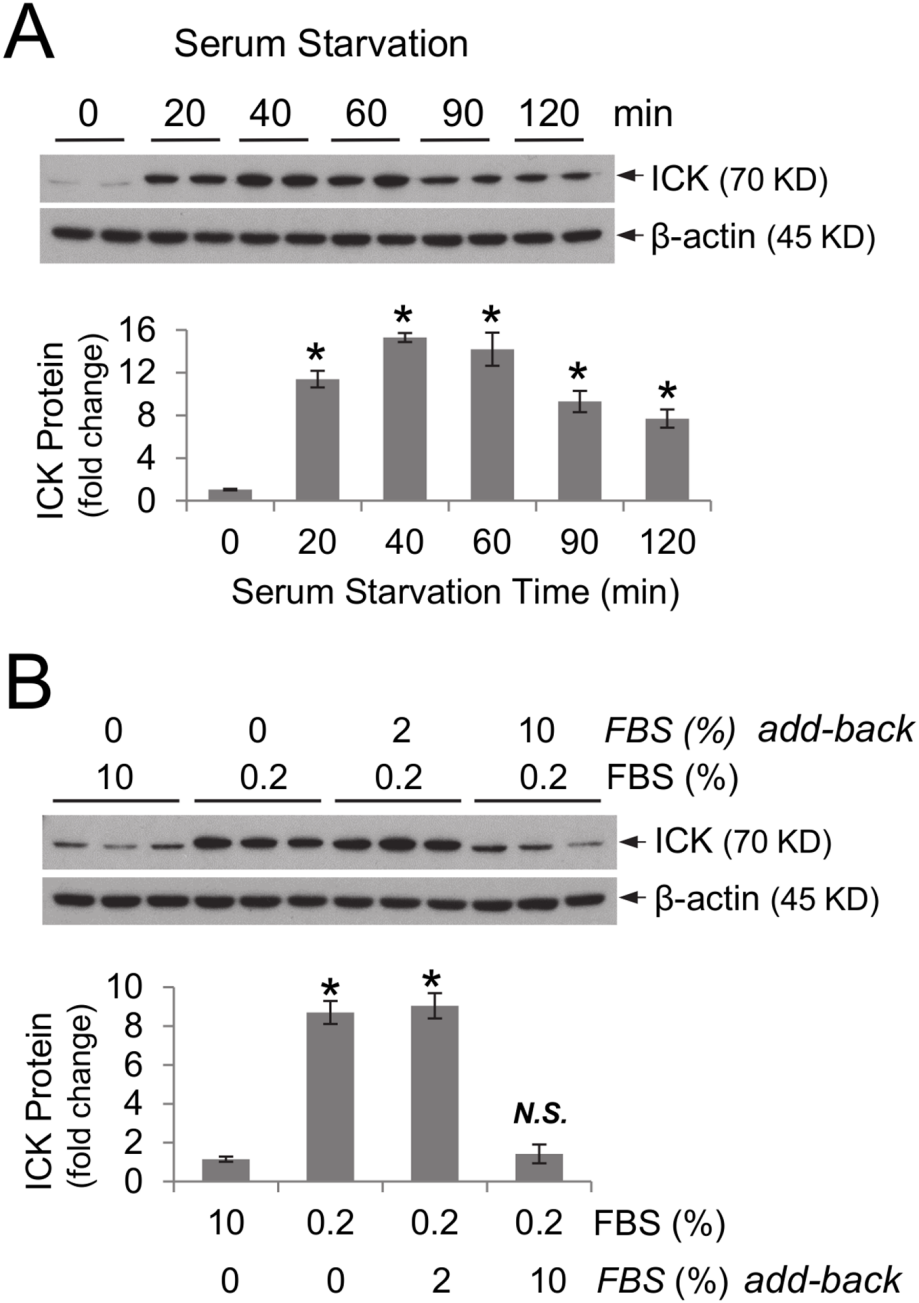
Serum-starvation of HCT-8 ileocecal epithelial cells *in* *vitro* induces acute up-regulation of ICK protein in a reversible manner. (A) HCT-8 cells were starved in growth medium containing 1% serum for various time points. Equal amount of total proteins from cell extracts were Western blotted against ICK and β-actin antibodies as indicated. After densitometry quantification and normalization against β-actin, the fold change of the ICK protein abundance relative to time zero during serum starvation was shown as mean ± SE, n = 2, *P<0.05. Similar results were obtained from three independent experiments. (B) HCT-8 cells were grown either in the complete medium containing 10% serum, or in the starvation medium containing 0.2% serum for 40 min, or in the starvation medium for 20 min followed by in the recovery medium containing either 2% or 10% serum for 20 min. Equal amount of total proteins from cell extracts were Western blotted against ICK and β-actin antibodies respectively. After densitometry quantification and normalization against β-actin, the fold change of the ICK protein level relative to the complete medium condition was shown as mean ± SE, n = 3, *P<0.05, *N.S.*, not significant. Similar results were obtained from three independent experiments.

Previously, we have established an *in* *vitro* model, the human ileocecal epithelial cell line HCT-8, to study the vicious cycle of malnutrition and infection [21]. We hereby used the HCT-8 model to explore the kinetics in nutritional regulation of ICK expression. In response to serum starvation, HCT-8 cells increased ICK protein abundance by about 10 fold within 20 min (Fig. 2A). This dramatic increase, albeit reduced in magnitude at later time points (Fig. 2A), persisted for at least 24 hours during serum starvation (data not shown). Adding back culture medium containing full serum (10% FBS) was able to quickly restore the basal level of ICK protein in HCT-8 cells (Fig. 2B). These data suggest that nutritional regulation of ICK protein abundance is an acute and reversible process.

### Serum albumin supplemented to the starvation medium only partially reversed starvation-induced effects on ICK protein

**Figure 3:**
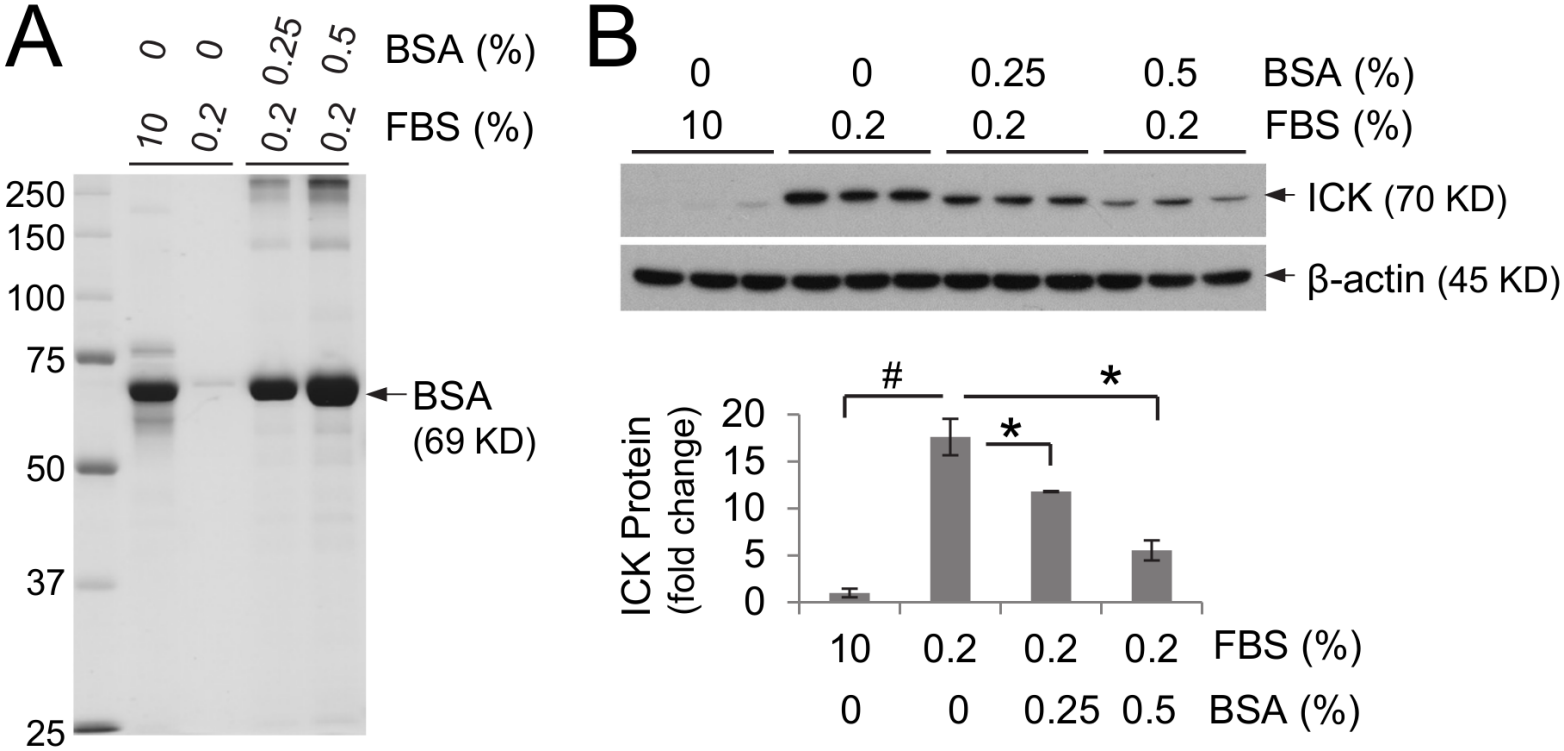
Serum albumin as a supplement to the starvation medium significantly lowered starvation-induced ICK protein level. (A) The albumin protein content from 1 µl of the complete medium (10% FBS), the starvation medium (0.2% FBS), or the starvation medium supplemented with either 0.25% or 0.5% BSA was analyzed and shown in a Coomassie blue-stained SDS-Gel. (B) HCT-8 cells were grown either in the complete medium containing 10% serum, or in the starvation medium containing 0.2% serum for 40 min, or in the starvation medium for 20 min followed by in the starvation medium supplemented with either 0.25% or 0.5% purified bovine serum albumin (BSA) for 20 min. Equal amount of total proteins from cell extracts were Western blotted against ICK and β-actin antibodies respectively. After densitometry quantification and normalization against β-actin, the fold change of the ICK protein level relative to the complete medium condition was shown as mean ± SE, n = 3, *P<0.05, ^#^P<0.01. Similar results were obtained from two independent experiments.

Since starvation, specifically omitting protein from diet, may cause a strong reduction in albumin production [22], we examined whether addition of purified albumin, the most abundant serum protein, in serum-starvation medium is able to restore the ICK protein level in HCT-8 cells. Addition of 0.25% purified bovine serum albumin (BSA) to the starvation medium containing 0.2% FBS was able to restore the amount of albumin found in the complete medium containing 10% FBS (Fig. 3A). In Figure 3B, the ICK protein level of cells in the starvation medium supplemented with 0.25% and 0.5% BSA decreased by about 30% and 60% respectively as compared with that of cells in the starvation medium alone. This observation implicated albumin as an important, but certainly not the only, factor in the serum that contributes to the regulation of ICK protein abundance. Interestingly, a similar effect on the regulation of ICK protein signals was observed when casein, a major nutrient protein in milk, was added as a supplement to the starvation medium (Fig. S1).

### Knockdown ICK expression in HCT-8 cells impaired cell proliferation and down-regulated the canonical Wnt pathway

**Figure 4:**
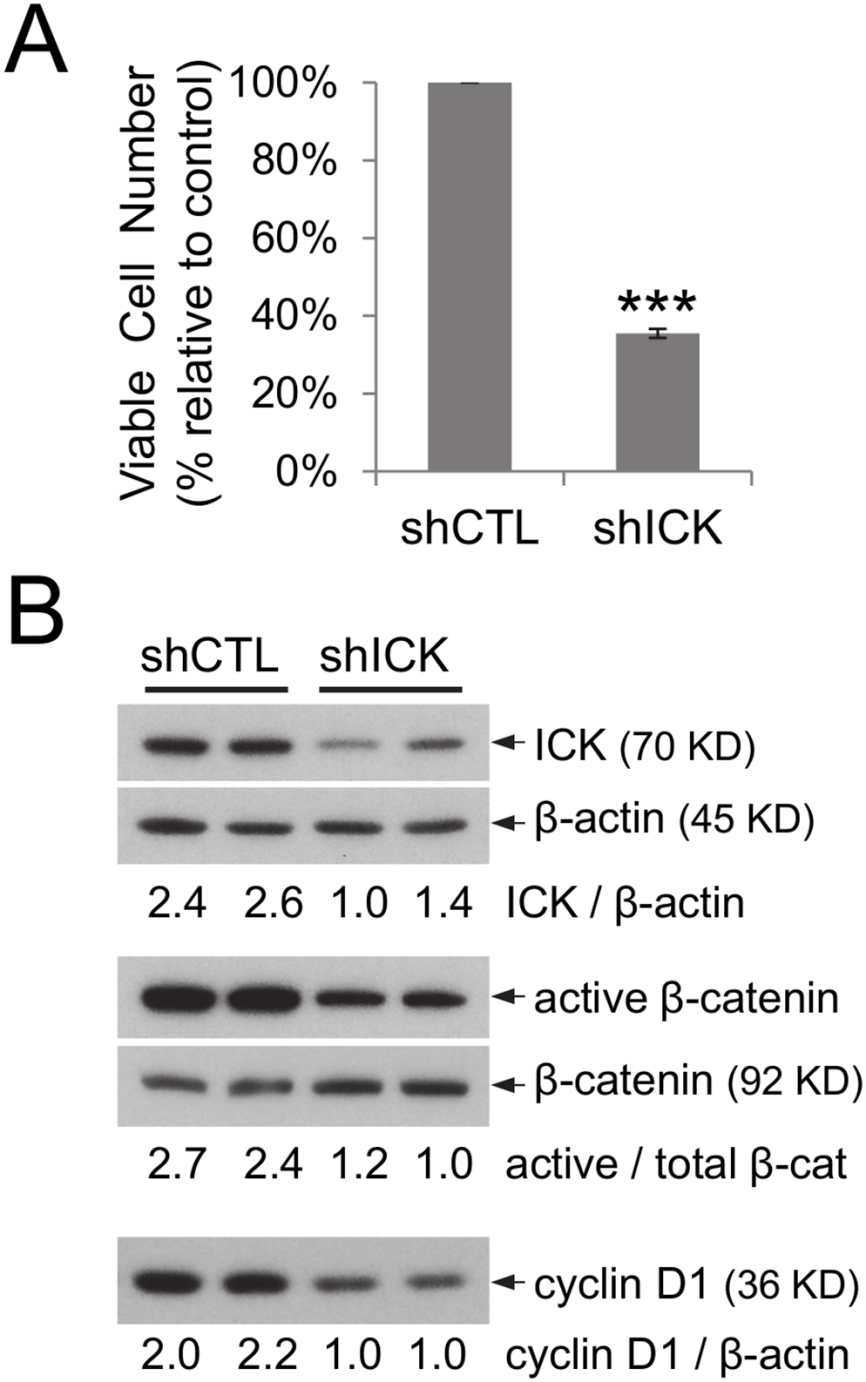
Reduced ICK expression in HCT-8 cells significantly impaired cell proliferation and down-regulated the Wnt/β-catenin pathway. (A) HCT-8 cells were infected with either the ICK-specific shRNA (shICK) or the control shRNA (shCTL). The total viable cell numbers were determined using trypan blue exclusion assay and shown as mean ± SE, n = 3, ***P<0.001. Similar results were obtained from two independent experiments. (B) Equal amount of total proteins from cell extracts was used on Western blot against antibodies recognizing ICK, β-actin, β-catenin, active β-catenin, and cyclin D1 as indicated. Western blot signals were quantified using densitometry and shown as the fold change after normalization.

Why do intestinal epithelial cells up-regulate ICK signals in response to nutritional stress? We hypothesized that elevated ICK signal is one of the protective mechanisms that intestinal cells utilize to promote compensatory cell growth and survival. To test this hypothesis, we infected HCT-8 cells using an ICK-targeted shRNA with validated specificity [10] and assessed the biological effects of ICK knockdown on cell proliferation and survival. Reduced ICK expression led to a 60% decrease in viable cell number (Fig. 4A), associated with a significant down-regulation of the active β-catenin signal and its downstream effector cyclin D1 (Fig. 4B), which was further confirmed by a second ICK-targeted shRNA (Fig. S2). These results indicate that ICK may regulate intestinal cell proliferation through functional interactions with the Wnt/β-catenin signaling events.

### ICK deficiency induced apoptosis by activating the caspase-dependent pathway

**Figure 5:**
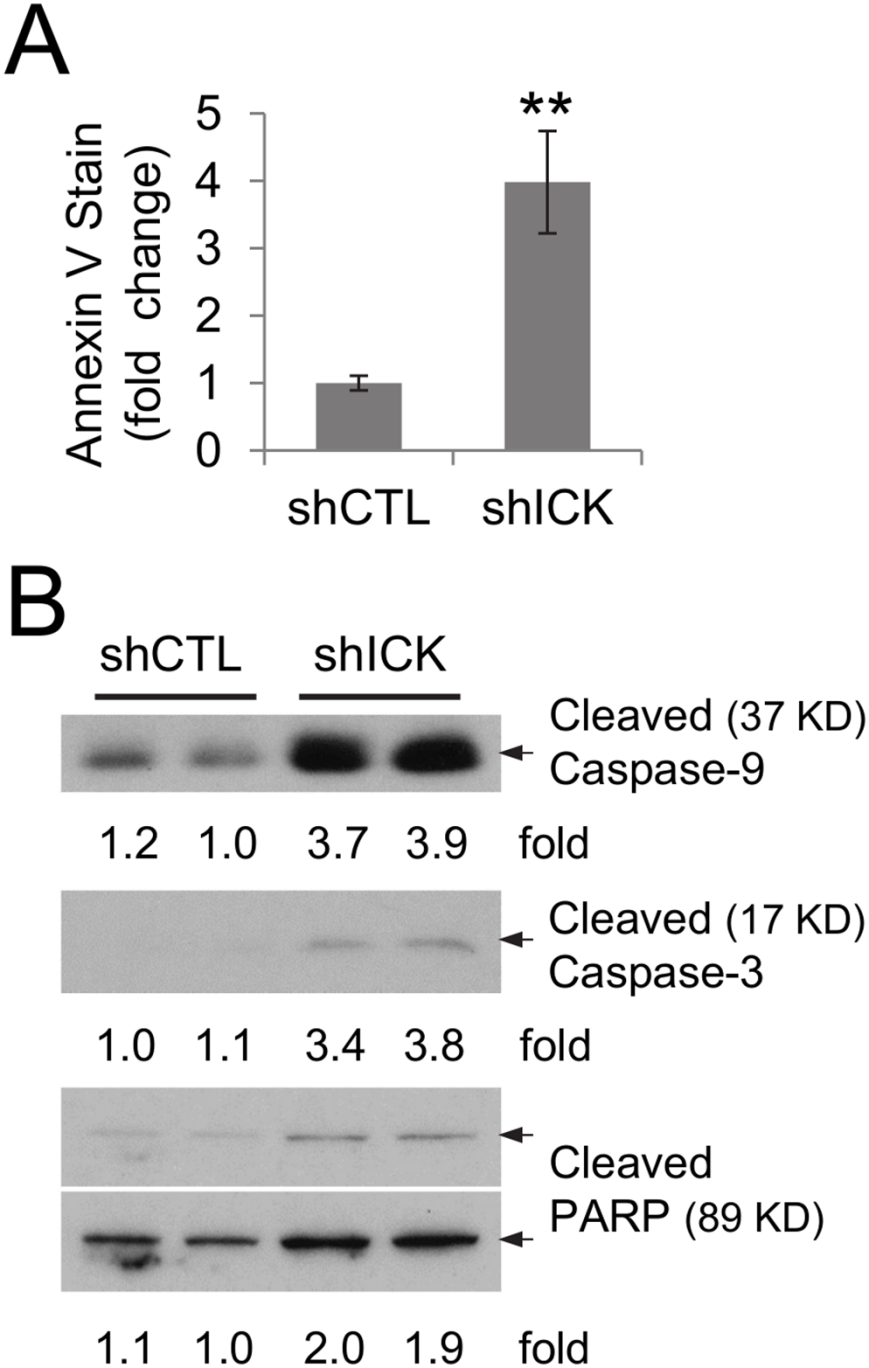
ICK deficiency in HCT-8 cells induced apoptosis via the caspase-dependent mechanism. (A) HCT-8 cells expressing either the ICK-specific shRNA (shICK) or the control shRNA (shCTL) were assessed for the number of apoptotic cells using Annexin V staining. The relative fold change of Annexin V stain-positive cells was shown as mean ± SE, n = 4, **P<0.01. Similar results were obtained from two independent experiments. (B) Equal amount of total proteins from cell extracts was used on Western blot against antibodies recognizing key components in the caspase pathway as indicated. Western blot signals were quantified using densitometry and shown as the fold change after normalization.

We also analyzed whether knockdown of ICK expression induces apoptosis that may contribute to the decreased intestinal cell viability. In Figure 5A, we demonstrated that HCT-8 cells expressing ICK-specific shRNA exhibited an approximately 4-fold increase in the number of apoptotic cells (detected by Annexin V staining) over HCT-8 cells expressing the control shRNA. This increased apoptosis induced by ICK knockdown was associated with a 2 to 4-fold increase in the expression of key components in the caspase pathway, such as cleaved caspase-9, cleaved caspase-3 and cleaved PARP (Fig. 5B). Our data suggest that ICK may promote intestinal cell survival by suppressing the caspase-dependent apoptotic pathway.

## Discussion

Nutritional deficiency could induce morphologic, biochemical, and metabolic alterations in humans and in experimental animal models [4], [23]. The small intestinal mucosa resists atrophy during protein deprivation for a longer time than most other tissues [24]. Major structural features of the intestinal mucosa, including villous height, crypt depth, epithelia cell number, and allocation of various cell lineages, are minimally, if at all altered (at least initially) by protein deficiency or starvation in experimental murine models [25], [26], [27], [28]. Mechanisms that may account for the preservation of intestinal architecture are not completely understood. Suppression of the normally high and rapid cellular turnover in the intestinal epithelium was observed during starvation [29], [30], implicating important roles for compensatory cell growth and survival in nutritional stress resistance. In this report, we provided novel mechanistic insights into the intestinal adaptation to protein malnutrition by showing robust but transient activation of multiple pro-proliferation and pro-survival pathways concomitant with elevated expression of intestinal stem cells.

The temporal and mechanistic modes of cell signaling responses to protein malnutrition appear to be diverse. Activation of Wnt/β-catenin, mTOR/S6K1, PI3K/Akt and CCRK/ICK pathways occurred in the early phase, at 24 hours of protein starvation, while activation of ERK and p38 MAPK pathways occurred in the late phase, at 72 hours of protein starvation. Compared with the transient activation of the Wnt/β-catenin, mTOR/S6K1, MAPKs, and ICK signaling pathways, activation of the PI3K/Akt pathway was sustained for a longer period of time. Mechanistically, activation of these pro-proliferation and pro-survival pathways occurred through either increased protein abundance (e.g. β-catenin, mTOR, ICK, Akt) or elevated phosphorylation of key regulatory sites (e.g. ERK1/2, p38 MAPK).

The rapid increase in ICK protein abundance induced by starvation is not due to enhanced transcription because our quantitative RT-PCR data did not reveal any significant change in the ICK mRNA level during starvation (data not shown). Since the small intestine has a very rapid rate of protein synthesis during normal nutrition and this rate is not markedly reduced during protein depletion [24], [31], it is likely that starvation-induced up-regulation of intestinal ICK protein content occurred as a result of increased protein synthesis and/or stability. One candidate gene that may be involved in the regulation of ICK protein degradation/stability is *FBXO9* (F-box only protein 9). *ICK* and *FBXO9* genes are arranged head-to-head on a bidirectional promoter [32]. It was recently discovered that FBXO9 has a functional role in adipocyte differentiation by serving as an ubiquitination E3 ligase to regulate C/EBPβ protein levels during adipogenesis [33]. Further studies will be aimed at investigating whether FBXO9 interacts with ICK to regulate its protein stability during nutrient starvation.

Our data showing that ICK functionally interacts with Wnt/β-catenin and caspase pathways provided novel insights into the molecular mechanisms by which ICK regulates intestinal cell proliferation and apoptosis. Currently, the direct downstream substrates of ICK that mediate its cross-talks with either the Wnt/β-catenin or the caspase pathway are still elusive. It is worthy of pointing out that ICK promoter contains functional binding sites for TCF4 and exogenously expressed β-catenin is able to robustly activate the ICK promoter activity *in* *vitro* [32]. Therefore, ICK may be a direct transcriptional target of the Wnt/β-catenin signal. Given our data in this study clearly indicated a negative impact of ICK knockdown on the signals of active β-catenin and its downstream effector cyclin D1, it is conceivable that there may exist a positive feedback regulatory loop between ICK and Wnt/β-catenin pathways.

In conclusion, our findings have provided novel insights into the molecular mechanisms regulating intestinal adaptation to protein nutritional stress. We have demonstrated that protein starvation induces strong but transient activation of prominent pro-proliferation and pro-survival pathways concurrent with elevated expression of intestinal stem cells. We also identified a novel signaling module involving functional cross-talks that ICK engages with Wnt/β-catenin and caspase pathways as an important participant in cell signaling responses to protein malnutrition. Cumulatively, these results suggest that activation of intestinal cell signaling pathways may serve as important molecular triggers for the cellular protective mechanisms underlying resistance to intestinal atrophy.

## Supporting Information

Figure S1 **Up-regulation of ICK protein induced by serum starvation in HCT-8 cells can be partially reversed by adding casein, a major nutrient source in milk, as the supplement.** HCT-8 cells were grown either in the complete medium containing 10% FBS or in the starvation medium containing 0.2% FBS for 40 min, or in the starvation medium supplemented with 0.25% and 0.5% casein for 20 min following starvation for 20 min. Equal amount of total proteins from cell extracts were Western blotted against ICK and β-actin antibodies respectively. After densitometry quantification and normalization against β-actin, the fold change of the ICK protein level relative to the complete medium condition was shown as mean ± SE, n = 3, *P<0.05, ^#^P<0.01. Similar results were obtained from two independent experiments.
(TIF)

Figure S2 **Knockdown of ICK in HCT-8 cells induced significant down-regulation of the active β-catenin signal as well as its downstream target cyclin D1.** HCT-8 cells were treated with either the control shRNA (shCTL) or the ICK-targeted shRNAs from Sigma MISSION as described in Methods. Equal amount of total proteins from cell extracts were Western blotted against antibodies as indicated. β-actin signal indicates equal loading of total proteins.
(TIF)
