# Supplementary figures and images for: Intestinal Cell Kinase Is a Novel Participant in Intestinal Cell Signaling Responses to Protein Malnutrition
Source: PLoS One. 2014 Sep 3;9(9):e106902. doi: 10.1371/journal.pone.0106902 (PMC4153720; doi:10.1371/journal.pone.0106902)

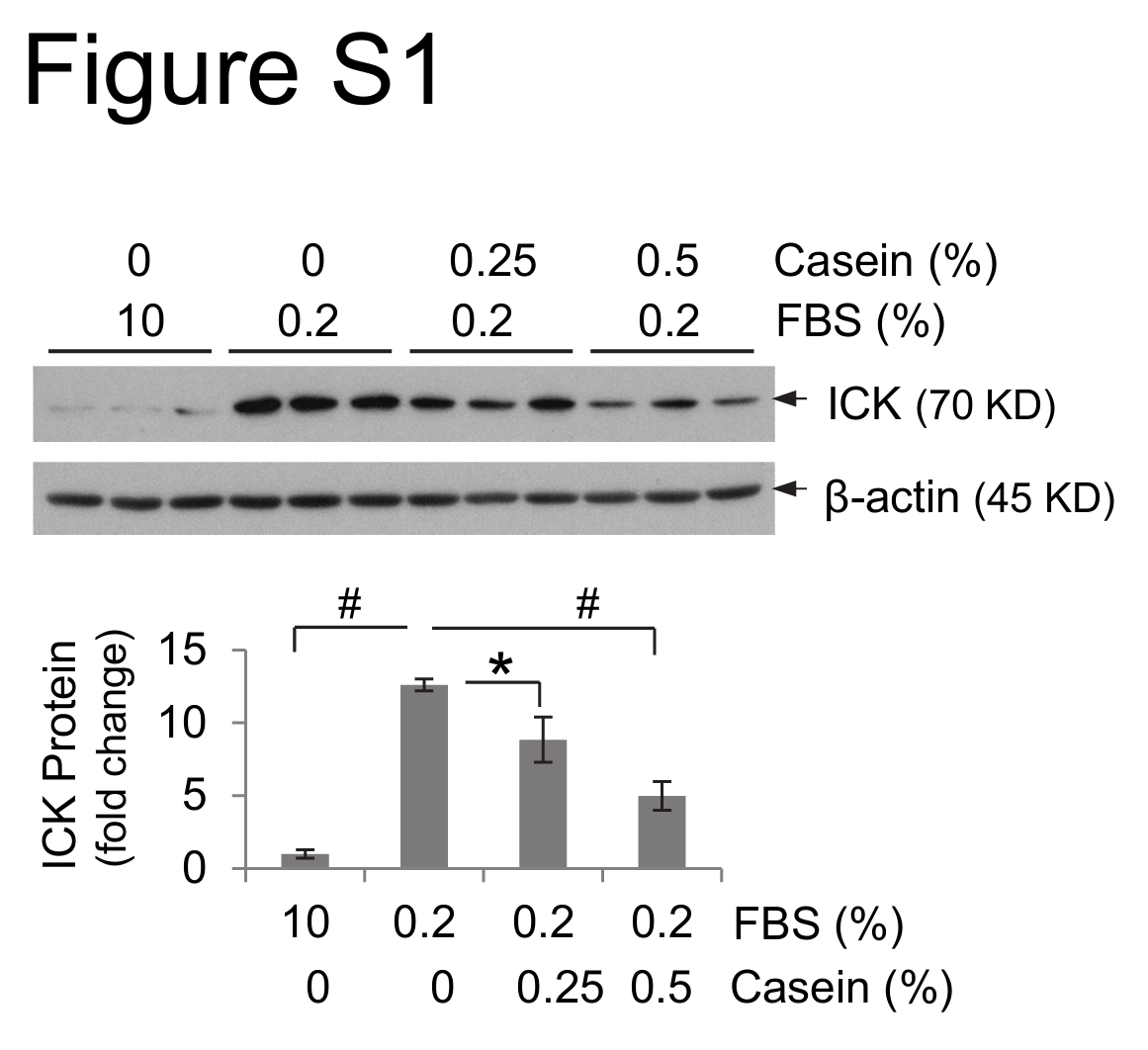

Supplement: Figure S1 — Up-regulation of ICK protein induced by serum starvation in HCT-8 cells can be partially reversed by adding casein, a major nutrient source in milk, as the supplement. HCT-8 cells were grown either in the complete medium containing 10% FBS or in the starvation medium containing 0.2% FBS for 40 min, or in the starvation medium supplemented with 0.25% and 0.5% casein for 20 min following starvation for 20 min. Equal amount of total proteins from cell extracts were Western blotted against ICK and β-actin antibodies respectively. After densitometry quantification and normalization against β-actin, the fold change of the ICK protein level relative to the complete medium condition was shown as mean ± SE, n = 3, *P<0.05, #P<0.01. Similar results were obtained from two independent experiments. (TIF) [file pone.0106902.s001.tif]

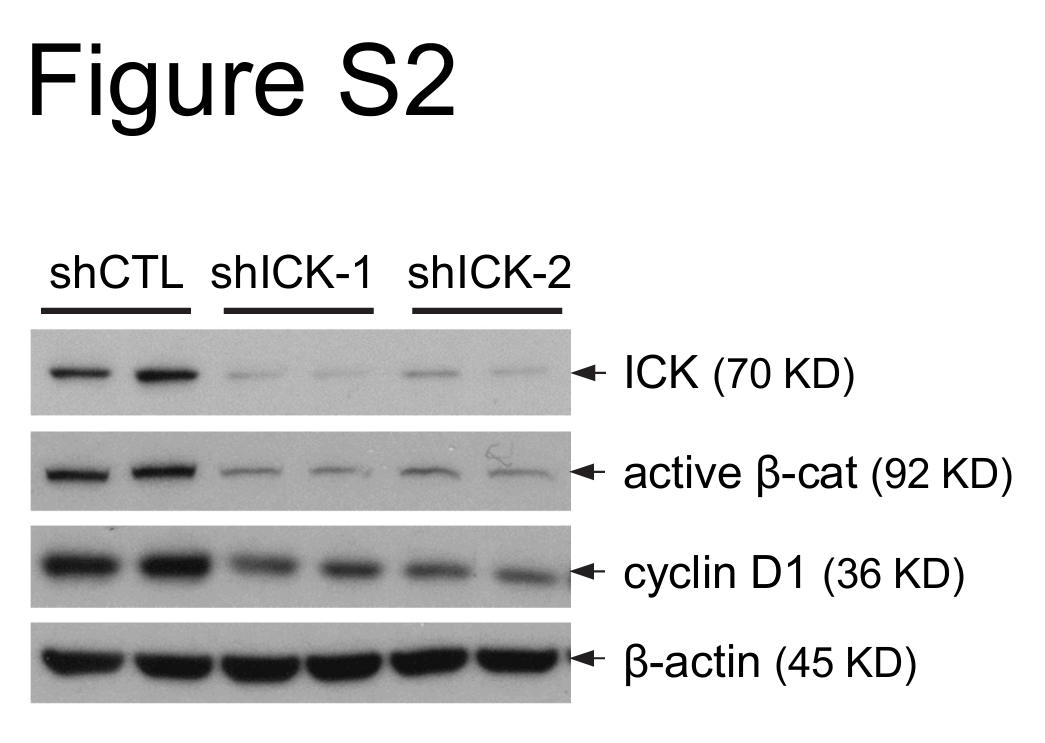

Supplement: Figure S2 — Knockdown of ICK in HCT-8 cells induced significant down-regulation of the active β-catenin signal as well as its downstream target cyclin D1. HCT-8 cells were treated with either the control shRNA (shCTL) or the ICK-targeted shRNAs from Sigma MISSION as described in Methods. Equal amount of total proteins from cell extracts were Western blotted against antibodies as indicated. β-actin signal indicates equal loading of total proteins. (TIF) [file pone.0106902.s002.tif]
